# Treatment of lead and arsenic poisoning in anuric patients – a case report and narrative review of the literature

**DOI:** 10.1186/s12882-019-1561-1

**Published:** 2019-10-17

**Authors:** Chun-Yuan Hsiao, Chip Gresham, Mark R. Marshall

**Affiliations:** 1 Department of Renal Medicine, Middlemore Hospital, Counties Manukau Health, Private Bag 93311, Otahuhu, Auckland, 1640 New Zealand; 2 Department of Emergency Medicine, Middlemore Hospital, Auckland, 1640 New Zealand; 3 National Poisons Centre, Dunedin, 9054 New Zealand; 4 Faculty of Medical and Health Sciences, University of Auckland, Auckland, 1142 New Zealand; 5 Baxter Healthcare (Asia Pacific) Pte Ltd, Singapore, 189673 Singapore

**Keywords:** Arsenic poisoning, Lead poisoning, Chelators, Anuria, Extracorporeal blood purification

## Abstract

**Background:**

Heavy metal poisoning can cause debilitating illness if left untreated, and its management in anuric patients poses challenges. Literature with which to guide clinical practice in this area is rather scattered.

**Case presentation:**

We present a case of symptomatic lead and arsenic poisoning from use of Ayurvedic medicine in a 28-year-old man with end-stage kidney disease on chronic hemodialysis. We describe his treatment course with chelating agents and extracorporeal blood purification, and review the relevant literature to provide general guidance.

**Conclusion:**

Cumulative clinical experience assists in identifying preferred chelators and modalities of extracorporeal blood purification when managing such patients. However, a larger body of real-world or clinical trial evidence is necessary to inform evidence-based guidelines for the management of heavy metal poisoning in anuric patients.

## Background

Heavy metal poisoning involving arsenic (Ars) and lead (Pb) can cause debilitation and death [1–3]. The major route of elimination of heavy metals is usually through the kidneys [1, 2], and in cases of poisoning this elimination can be enhanced by chelating agents [4–7]. The most common agents are dimercaprol, also known as British Anti-Lewisite (BAL); calcium-disodium-ethylenediaminetetraacetic acid (CaNa_2_EDTA); dimercaptosuccinic acid, also known as succinmer (DMSA); and 2,3-Dimercapto-1-propanesulfonate (DMPS). So long as renal function is not severely impaired, treatment of poisoning does not normally require adjunctive extracorporeal blood purification (EBP), which is inferior to removal of heavy metals by normally functioning kidneys [8–11].

In patients who are anuric, whether from acute kidney injury (AKI) or end-stage kidney failure (ESKF), EBP is the only means for removal of heavy metals, and a critical intervention alongside general measures to support and preserve organ function. When using EBP, chelating agents are generally mandatory to achieve rapid removal and meaningful detoxification. Although these general principles are well known, there is only a small published literature reporting a wide range of practices often without clear links to outcomes. For a clinician facing such a case in clinical practice, choosing the right chelating agent for a given patient can be daunting. We describe an instructive case and review the relevant literature, thereby providing general guidance on the timing, type, dose and duration of both chelation therapy during EBP for heavy metal poisoning in anuric patients.

## Case presentation

A 28-year-old man on maintenance hemodialysis (HD) was diagnosed with steroid-resistant primary focal segmental glomerulosclerosis causing nephrotic syndrome in February 2006. There was only a partial response to ciclosporin, and he reached end-stage kidney failure requiring hemodialysis in August 2011. He presented with progressive global motor weakness, tremor and hallucinations in December 2011. This was preceded by eight months of polyarthralgia, constipation, abdominal pain, and nausea and vomiting, and one month of severe neuropathic pain in all limbs, nightmares and involuntary vocalization. To enquiry, he admitted taking Ayurvedic medicine over a long but unquantifiable duration.

Physical examination revealed a blood pressure of 140/90 mmHg, a heart rate of 76 beats per minute and a respiratory rate of 18 per minute. His cardiovascular, respiratory, abdominal and joint examinations were unremarkable. Alopecia was notable, although we could not identify any skin or nail abnormalities. The striking neurological findings were moderate-to-severe muscle weakness involving the face, neck and all limbs; absent reflexes at C6, C7 and S1; impaired proprioception in the hands and feet; and a patchy disturbance of light touch and pinprick over all limbs.

He had normocytic normochromic anemia, with erythrocyte basophilic stippling noted on two of his numerous peripheral smears. His erythrocyte porphyrins level was 5 umol/L (normal < 1.8 umol/L). Nerve conduction studies revealed axonal sensorimotor polyradiculoneuropathy. Other investigations included magnetic resonance imaging of brain, cerebral spinal fluid analysis, autoimmune, infection and metabolic screens, and were all unremarkable.

At this point, a provisional diagnosis of heavy metal poisoning was considered, based upon his history of Ayurvedic medicine exposure. His blood Pb level was found to be significantly elevated at 6.3 umol/L (normal < 0.47 umol/L), but his blood mercury level was normal at 5 nmol/L (normal < 50 nmol/L). His blood was not tested for Ars, although pooled (scalp, chest and arms) hair samples were sent for testing at this time. Parathyroid hormone level was 7.5 pmol/L (normal 1.7 – 7.3 pmol/L). The ayurvedic products recovered from the patient’s domicile contained high levels of Ars (50 to 290 mg/kg), Pb (28 to 12,000 mg/kg) and mercury (5 to 75,000 mg/kg).

Due to initial unavailability of CaNa_2_EDTA and DMPS, we treated him for 5 days with continuous veno-venous haemodiafiltration (CVVHDF) and BAL administered intramuscularly at 4 mg/kg every 4 h. When CaNa_2_EDTA became available, we switched him to thrice weekly standard high-flux HD with the 1 g of CaNa_2_EDTA administered over 1 h, given 1 – 3 h before HD. His nightmares, involuntary vocalisation and gastrointestinal symptoms resolved over a few weeks, although his peripheral neuropathy and alopecia persisted with only mild improvement. After a month, the results of initial testing for Ars became available. The level in his hair samples was 1.21 mg/kg (normal < 0.1 mg/kg), suggesting ongoing exposure in the 2 to 3 months prior to hospital admission. Our assessment was that the residual peripheral neuropathy was most likely attributable to Ars, as it tends to cause a sensorimotor polyneuropathy often presenting with predominantly sensory symptoms, while Pb poisoning usually presents with minimal to no sensory involvement [3, 12]. On the basis of this assessment, we felt that it was reasonable to attempt a course of chelation specifically for Ars. We chose DMPS on the basis of its favourable toxicity profile, which was administered to him orally 2 – 3 h before each HD. We discontinued this medication after 2 weeks, however, due to a lack of clinical improvement and evidence of mobilisation of Ars, with levels that remained consistently undetectable in both blood and dialysate (< 0.05 umol/L).

**Fig. 1:**
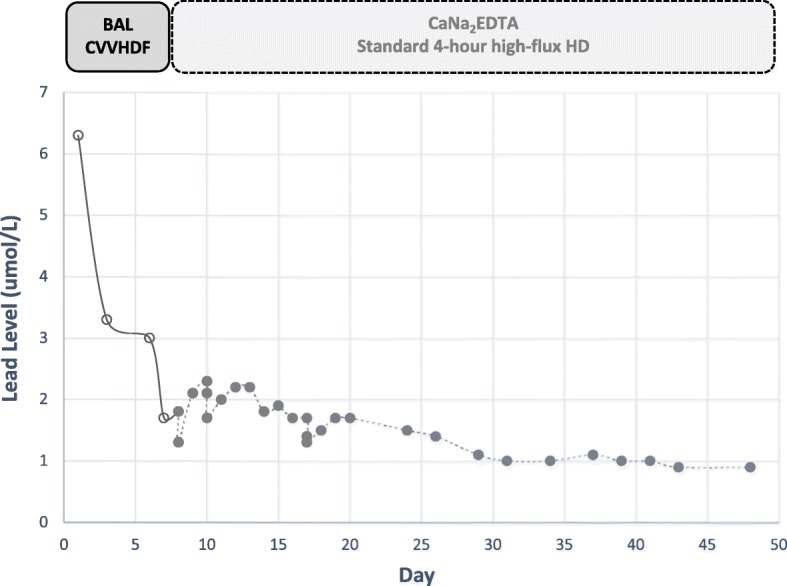
Blood lead concentration-time graph in relation to the use of different chelating agents and different modalities of extracorporeal tblood purification. Solid line - BAL (4 mg/kg IM every 4 h) with continuous venovenous hemodiafiltration (CVVHDF); dashed line - CaNa_2_EDTA (1 g IV) given 1 to 3 h before 4-h high-flux hemodialysis (HD)

In total, we provided 8 weeks of Pb chelation, after which time the blood Pb level decreased and remained less than 1.2 umol/L (see Fig. 1). Over the following 8 months of follow up, there was only mild improvement of his tremors and weakness, and the peripheral neuropathy persisted, presumably on the basis of chronic arsenicosis. There were no chelator-associated adverse effects during the course of the treatment.

## Discussion and conclusions

### Toxicokinetics of Lead and arsenic

Pb and Ars are heavy metals with low atomic weights [2, 13], but despite this are not rapidly removed by EBP without chelation due to other toxicokinetic properties. With Pb, ~ 99% binds to erythrocytes within the first hour, following which there is rapid distribution in the body with binding to sulfhydryl and carboxyl groups on a wide variety of structural and functional proteins in the central and peripheral nervous, cardiovascular, renal, reproductive, musculoskeletal, hematopoietic, and other organ systems. Over time, ~ 95% of total body Pb burden is stored in bone [2]. These properties reduce the availability Pb for extracorporeal removal without a chelator.

Ars has a low serum protein binding ranging from 5.3% (inorganic trivalent arsenic) to 6.5% (inorganic pentavalent arsenic) [13], but a high volume of distribution (3.3 to 4 L/kg) and rapid redistribution from serum to tissues (phase 1 half-life 1-2 h, with 90% of Ars redistributing within 3 h) [14]. Theoretically, then, there is a therapeutic window for early EBP without chelation in first few hours after ingestion. For instance, in a case of massive Ars poisoning with anuric AKI, one group used high-flux HD for 4 h without a chelator, achieving an extracorporeal Ars clearance of 85 ml/min and removal of 8 mg of Ars [10]. In another report, a similar regimen was shown to remove approximately 67% of serum Ars, albeit in chronic HD patients who were not poisoned with normal background serum arsenic levels averaging 6.47 μg/l [15]. Despite these reports, a chelator is strongly recommended for all stages of acute Ars poisoning in anuric patients, since it will always enhance availability of the metal in serum for dialysis [10], and minimise chances of subsequent morbidity and mortality.

### Chelation therapy

It is important to select an appropriate chelator that is suitable for treatment of the specific poisoning, and dosed correctly to minimise chelator-induced adverse effects. In the setting of anuria, there is also accumulation of the chelator-metal complex, and without removal (by either by native kidneys or EBP) this will lead to redistribution of the metal into central nervous system. As such, EBP should be promptly administered with chelation for optimal removal of both chelated and non-chelated metals. In addition, it should be remembered that poisoned patients with AKI invariably develop metabolic acidosis, and this may decrease the effectiveness of chelators. Tables 1, 2 and 3 summarise the pharmacologic basis of the chelating agents, and the literature describing their use in treating Pb and Ars poisoning. Adverse effects are generally dose related and a non-exhaustive list is included in Table 1.

**Table 1:** Chemical properties and pharmacologic basis of the chelating agents used in lead and arsenic poisoning

	BAL	DMPS	CaNa_2_EDTA	DMSA
Amendable to EBP	+	++	+++	+ / -
Molecular Weight (Dalton)	124	228	374	182
Chelate-Pb Complex MW (Dalton) ^a^	331 (455 if ratio is 2:1) [3]	434	540	389
Chelate-Ars Complex MW (Dalton) ^a^	199 (323 if ratio is 2:1) [3]	303 (803 if ratio is 3:2) [4]	Cannot chelate arsenic [16]	257
Administration Route	IM in peanut oil medium	PO, IV	IV, IP, IM	PO
Distribution	Lipophilic	Hydrophilic	Hydrophilic	Hydrophilic
Volume of Distribution	High	0.16 L/kg [4]	0.21 L/kg [17]	0.4 L/kg ^b^ [18]
Protein Binding	n/a	62.5% [19]	11 – 19% [20]	95% [21]
Excretion	Renal (Major)	Renal (46 – 59%) Biliary (extent undetermined)	Renal (Major)	Renal (Major)
LD50 (mmol/kg)	1.48 ^c^ [16]	6.53 ^c^ [16]	16.4 [5]	13.73 ^c^ [16]
Contraindications	Peanut allergies, hepatic dysfunction, methylmercury poisoning	None in acute lead or arsenic toxicity	None in acute lead toxicity	None in acute lead or arsenic toxicity
Adverse Effects	Nausea, vomiting, headache, hypertension, pain and/or sterile abscess at injection site, haemolysis in G6PD-deficiency, chelation of essential metals in prolonged use	Allergic reaction, nausea, vomiting, Steven-Johnson syndrome (rare)	Fatigue, headache, mild AST/ALT elevations, nephrotoxicity, chelation of essential metals in prolonged use	Nausea, vomiting, diarrhoea, mild AST/ALT elevations, Fever, rash, reversible neutropenia (rare), chelation of essential metals in prolonged use

*MW* molecular weight, *IM* intramuscular, *IV* intravenous, *PO* oral, *IP* intraperitoneal, *Pb* lead, *Ars* arsenic

^a^: the ratio of chelate-metal complex is presumably 1:1 as data is limited; n/a: not available

^b^: based on primates

^c^: based on mice IP

**Table 2:** Extracorporeal removal of lead by different modes of extracorporeal blood purification in patients with acute or chronic lead intoxication after an initial administration of intravenous 500 mg to 1000 mg of CaNa_2_EDTA

Patients	Renal Function	Mode of Dialytic Therapy	Initial Blood Pb Level	Post-dialysis Blood Pb Level	Dialytic Pb Removal	Urinary Pb Excretion	Outcome
1 – Smith [37]	Normal	HD - 2 h	3.1 mg/g	1.0 mg/g	3.4 mg	n/a	Died
2	Normal	HD - 2 h	2.7 mg/g	1.3 mg/g	3.0 mg	n/a	Improved encephalopathy
3	Normal	HD - 30 min	1.6 mg/g	1.1 mg/g	1.0 mg	n/a	Remained severely encephalopathic
4	Normal	HD - 2 h	1.8 mg/g	0.7 mg/g	2.2 mg	n/a	Died
1 – Mehbod [38]	GFR < 10	CAPD	n/a	n/a	16.9 mg/20 h	0.12 mg/20 h	Weakness; constipation and anaemia improved
2	Normal	CAPD	n/a	n/a	1.90 mg/20 h	0.50 mg/20 h	No immediate improvement; no long-term follow up
3	Normal	CAPD	n/a	n/a	1.48 mg/20 h	0.70 mg/20 h	No immediate improvement; no long-term follow up
4	Normal	CAPD	1.25 mg/L	0.75 mg/L	2.00 mg/20 h	0.40 mg/20 h	Improved encephalopathy; no long-term follow up
1 – Pedersen [39]	Normal	HD – 9 h	1.36 mg/L	0.68 mg/L	n/a	n/a	Serum Pb level 0.28 mg/L after 7 weeks; no clinical outcome reported
1 – Roger [40]	ESKF	CAPD	1.40 μmol/L	n/a	9.29 μmol over 4 days	2.96 μmol/day on day 4	Improved mental state but peripheral neuropathy progressed after 4 months
1 – Kessler [41]	ESKF	HF	0.279 mg/L	n/a	1.650 mg/day ^a^		n/a
2	ESKF	HF	0.131 mg/L	n/a	1.450 mg/day ^a^		n/a
3	ESKF	HF	0.361 mg/L	n/a	1.152 mg/day ^a^		Died
4	ESKF	HF	0.281 mg/L	n/a	1.267 mg/day ^a^		n/a
5	ESKF	CAPD	0.265 mg/L	n/a	0.334 mg/day	0.476 mg/day	n/a
1 – Kessler [42]	ESKF	HF	0.280 mg/L	n/a	3.300 mg/day ^a^		n/a
2	ESKF	CAPD	0.265 mg/L	n/a	0.710 mg/day ^a^		n/a
1 – Barats [43]	ESKF	HD	0.690 mg/L	0.110 mg/L^b^	n/a	n/a	Resolved encephalopathy and motor neuropathy
1 – Roberts [44]	ESKF	HD	0.49 μg/L	n/a	0.24 mg	0.025 mg	Lead mobilisation test

*ESKF* end-stage kidney failure, *GFR* glomerular filtration rate, *HD* haemodialysis, *HF* haemofiltration, *CAPD* continuous ambulatory peritoneal dialysis, *Pb* lead

^a^: combined urinary removal of lead over 24 h and dialysis; ^b^: 3 months after chelation; n/a: not available

**Table 3:** Effectiveness of various modes of extracorporeal blood purification and different chelators in arsenic intoxication

Patients	Renal Function (Creatinine)	Pre-dialysis Ars Level	Post-dialysis Ars Level	Chelator	Mode of Dialytic Therapy	Dialytic Ars Clearance	Dialytic Ars Removal	Urinary Ars Clearance	Urinary Ars Removal	Outcome
Giberson [24]	288 μmol/L Oliguric	300 μg/L	140 μg/L	BAL	HD 4 h	87 ml/min	3.36 mg over 4 h	n/a	2.03 mg/day	Resolved GI symptoms and renal function recovered
Vaziri [8]	707 μmol/L	411 μg/L	364 μg/L	BAL	HD 4 h	76 ml/min	4.68 mg over 4 h	n/a	3.12 mg/day	Resolved GI symptoms and renal function recovered
Smith [26]	Oliguric	26.7 μg/L	29.0 μg/L	BAL and Penicillamine	HP 2 h	n/a	n/a			Improved renal function and remained comatosed after 100 days
		29.0 μg/L	36.2 μg/L		HD 2 h					
		21.7 μg/L	22.7 μg/L		HD 3 h					
		18.9 μg/L	21.5 μg/L		HP 3 h					
Levin-Scherz [27]	495 μmol/L Anuric	190 μg/L	62 μg/L	BAL	HD 4 h	n/a	2.9 mg over 4 h	n/a	n/a	Died
Mathieu [10]	238 μmol/L Anuric^a^	249 μg/L	133 μg/L	No chelator	HD 4 h	85 ml/min	8.2 mg over 4 h	n/a	10.7 mg	Renal function recovered
		110 μg/L	91 μg/L	BAL	HD 4 h	87.5 ml/min	5.3 mg over 4 h	n/a	13.2 mg	
1 – Blyth [25]	Oliguric^a^	80 μg/L	n/a	BAL	HD			358 ml/min	94 mg/72 h	Renal function recovered
2	Oliguric	340 μg/L	n/a	BAL	CVVH	15 ml/min	23 mg over 90 h	3.83 ml/min	6 mg/99 h	Died
Fesmir [28]	Normal	444 μg/L	170 μg/L	BAL and Penicillamine	HD	n/a	n/a	n/a	24 mg/day	Developed PN and HT
Hanston [29]	459 μmol/L Oliguric	n/a	n/a	BAL + DMSA IV 2 days → DMSA IV 9 days	HD	37.5 ml/min	26 mg over 11 days	1.34 ml/min	15 mg over 11 days	Died
					CVVH	0.64 ml/min	8 mg over 11 days			
				DMSA IP 11 days	PD	4.28 ml/min	17 mg over 11 days			
Zilker [11]	Normal	245 μg/L	2.3 μg/L^b^	DMPS	HD 5 h	n/a	0.168 mg	n/a	89.67 mg over 87 h	Recovered
					CAVHDF		0.061 mg			
					HD 5 h		0.12 mg			
Lai [14]	Anuric	730 μg/L at autopsy	n/a	BAL DMPS	HD	n/a	2.04 mg			Died
1 – Adam [30]	406 μmol/L Oliguric	558 μg/L	n/a	BAL	HD-HP 3.5 – 4 h	55.7 – 70.4 ml/min	3.8 – 5.2 mg per session	37.1 ml/min	156 mg over 20 days	Paraplegic
					PD		2.83 mg over 6 days			
2	247 μmol/L Oliguric	540 μg/L	n/a	BAL	HD-HP 3.5 h	125.8 ml/min	11.62 mg per session		8.54 mg over 34 h	Died
3	274 μmol/L Oliguric	620 μg/L	n/a	BAL DMPS	HD-HP 3.5 h	47.3 – 54 ml/min	2.28 – 2.89 mg per session	68 ml/min	123.4 mg over 4 days	Died
4	Normal	245 μg/L	n/a	DMPS	HD 5 h	40 ml/min	0.24 mg	696 ml/min	86.25 mg over 3 days	Fully recovered
					CAVHDF	32.1 – 54.7 ml/min	0.063 mg over 6 h and 0.051 mg over 12 h			
5	Normal	2240 μg/L	n/a	DMPS	Nil	n/a	n/a	183 - 436 ml/min	134.6 mg over 4 days	Fully recovered
6	901 μmol/L Anuric	4469 μg/L	n/a	DMPS	HDF 5 h	42.2 - 140.3 (average 98) ml/min	252 mg over 6 sessions		Anuric	Fully recovered

*Ars* arsenic, *PN* peripheral neuropathy, *HT* hypertension, *GI* gastrointestinal, *n/a* not available, *HD* haemodialysis, *CVVH* continuous veno-venous haemofiltration, *HDF* haemodiafiltration, *HD-HP* haemodialysis-haemoperfusion, *CAVHDF* continuous arterio-venous haemodiafiltration, *IV* intravenous, *IP* intraperitoneal

^a^: renal function recovered in hours after commencement of treatment; ^b^: serum arsenic level after 7 days

### Dimercaprol (BAL)

BAL is a chelator originally used in Ars poisoning, Nowadays, it is most commonly used in conjunction with CaNa_2_EDTA for severe Pb poisoning or Pb-induced encephalopathy, where it is administered 4 h prior to giving CaNa_2_EDTA (see section on CaNa_2_EDTA below). BAL itself has a low molecular weight of 124 Da, and BAL-Pb and BAL-Ars chelated complexes have molecular weights of 455 Da and 323 Da, respectively, assuming a stable (though not well described) BAL-metal 2:1 ratio [3]. Data on protein binding in plasma are not available for BAL and its metabolites, or for protein binding and volume of distribution of BAL-Pb and BAL-Ars compounds.

BAL is administered intramuscularly in peanut oil [3]. Of note, the drug itself has a poor toxicity profile compared to other chelators. With doses of 4 mg/kg and 5 mg/kg, the incidence of reported adverse effects is as high as 14 to 65%, respectively [22]. This risk is increased in anuria due to accumulation. BAL does undergo a degree of hepatic glucuronidation forming glucuronic acid conjugates [22]. Theoretically, these metabolites are more hydrophilic than BAL, and thus more easily removed by kidneys and presumably EBP [23]. Notwithstanding, there is still a greater risk of adverse effects from BAL in the setting of anuria.

Literature describing ideal prescription of EBP with concurrent BAL use is limited, but longer EBP treatments theoretically allow for greater removal of the metal-chelator complex. As BAL reaches its peak concentration in serum approximately 30 to 60 min after intramuscular injection [3]. EBP should be performed within one hour after the administration of BAL.

There is little reported experience with BAL as a sole Pb chelator with EBP in setting of anuria. In the case presented in this research, we only used BAL because of the initial unavailability of CaNa_2_EDTA and DMPS. Notwithstanding, we showed that CVVHDF was effective in removing BAL-Pb (Fig. 1), allowing us to use the full recommended dose of BAL, whilst minimising the risk of blood Pb rebound that is usually seen after intermittent HD, as well as the risk of significant accumulation of the chelator in the body. Despite our positive experience, we believe BAL should not be used as a sole chelator in anuric patients if other options are available.

There are several reports of using BAL with EBP for acute Ars poisoning in the setting of impaired kidney function. In two anuric patients treated with BAL, Ars removal was between 50 and 100% greater with even low flux-HD compared to that removed by their residual kidney function, consistently removing between 2 and 6 mg of Ars per treatment [8, 24]. With more modest degrees of renal impairment, Ars removal with HD is still considerable, but less important than native kidney removal. For instance, one report described 10.7 mg of Ars eliminated by the kidneys over 24 h in a patient with a serum creatinine of 27 mg/L, compared to only 8.2 mg eliminated by a 4 h high flux HD session [10]. Importantly, the efficacy of Ars removal with EBP is often expressed as clearance, and which is sometimes not increased by BAL [8, 10, 24, 25]. This should not be misinterpreted as showing limited efficacy – in all cases, BAL increased the serum concentration of Ars available to EBP per unit time, leading to greater removal (i.e. when expressed in mass balance terms) despite similar clearance.

The reported use of BAL with EBP for acute Ars poisoning is summarized in Table 3 [14, 25–28]. The potential benefits of BAL in this setting needs to be carefully weighed against the risks, which arise from its relatively greater lipophilicity compared to DMSA and DMPS. This can lead to abrupt mobilization of Ars from tissue, and redistribution into central nervous system with acute exacerbation of neuro-encephalopathy [31]. To our knowledge, this complication has not yet been reported in the literature in the clinical setting, this is primarily because of careful measures that are universally taken to avoid its occurrence. In our patient, any such effect was obviated by EBP in the form of CVVHDF immediately after administration of BAL continuously for numerous days, providing prompt and uninterrupted removal of mobilized Ars thereby avoiding accumulation and post-dialysis rebound. Given that the diagnosis of Ars poisoning was delayed in our case, it was reassuring that we detected no clinical deterioration in our patient with BAL and CVVHDF, even though we did not look for evidence of mobilisation of Ars at the time.

The benefit of chelators in chronic arsenicosis is debated, but generally not recommended as most health effects in this condition are irreversible. In support of this, a small randomised-placebo controlled trial involving 22 patients showed BAL to be ineffective for this condition [32], and there was no significant improvement in our patient’s peripheral neuropathy after chelation for several weeks.

### Dimercaptosuccinic acid (DMSA/Succimer)

DMSA is an orally-administered water-soluble analogue of BAL that has a higher therapeutic index. It is one of the chelating agents of choice for acute and chronic Pb poisoning in patients with normal kidney function. DMSA can also be considered for the treatment acute Ars poisoning.

DMSA has a low molecular weight of 182 Da, and a low volume of distribution of approximately 0.4 L/kg [18]. The molecular weights of DMSA-Pb and DMSA-Ars complex are 389 Da and 257 Da, respectively, based on the 1:1 DMSA-metal complex ratio. However, DMSA is 95% protein-bound in plasma [21], although there are no data on protein binding of DMSA-metal complexes. This makes DMSA (and probably its DMSA-metal complex) unfavourable for extracorporeal removal. Adding more limitations to the use DMSA in anuric patients is that DMSA appears to be a prodrug in humans. It conjugates with cysteine to form DMSA-cysteine disulfides as an active chelator by an unclear mechanism in kidney’s proximal tubular cells [33]. This important process is unlikely to occur in completely impaired kidneys. Interestingly, DMSA-cysteine disulfides is not formed in mice, rats and rabbits, and the unaltered DMSA is able to chelate Pb to some degree in these species [33].

There is limited literature describing the use of DMSA with EBP. Though hemoperfusion is useful in removing highly protein-bound molecules [34], the affinity of DMSA and its chelated complex to the activated charcoal or resin cartridge is unknown, and to our knowledge there is no reported experience in human subjects with Pb and Ars poisoning. There are other practical disadvantages to hemoperfusion, including the need for frequent change of cartridges due to rapid saturation, the limited shelf-life of the cartridges, the lack of clinical experience with this form of due to infrequent use, and the generally high cost [34, 35]. Hantson et al reported generally low extracorporeal Ars removal using intravenous DMSA and other EBP techniques in a 26-year-old oliguric patient who probably had 10 g of arsenic trioxide ingestion over 2 weeks [29]. The extracorporeal Ars clearance was 0.64 ml/min for continuous veno-venous haemofiltration (CVVH), 4.28 ml/min for peritoneal dialysis (PD) and 37.51 ml/min for HD. Sheabar et al showed in vitro that DMSA actually inhibited Ars removal across a standard dialysis membrane, but not across a dialysis membrane with molecular weight cut-off (MWCO) of 12,000 – 14,000 [36]. Presumably, the large protein-chelator-metal complexes are unable to cross a standard dialysis membrane, but able to cross a more porous one.

Because of our patient’s encephalopathy, and the limited evidence and experience with DMSA, we chose BAL (both drugs were available to us at the time) and continued with BAL until his encephalopathy resolved and CaNa_2_EDTA became available. DMSA with or without EBP in anuric patients with Pb or Ars poisoning should probably only be considered if no other options are available, and then only with a high MWCO dialysis membrane.

### Calcium-disodium Edetate (CaNa_2_EDTA)

CaNa_2_EDTA is one of the chelating agents of choice in Pb poisoning for its effective Pb chelation, high therapeutic index, and good cumulative clinical experience of favourable outcomes as summarized in Table 2. It is usually given intravenously but can be administered intramuscularly and intraperitoneally. The chelator has potential to cause AKI as a direct adverse event, and this risk increases at doses of more than 75 mg/kg/day [5]. Other disadvantages of this agent are its poor oral bioavailability at less than 5%, and its associated risk of depletion of essential metals such as iron, zinc and manganese [3].

Combining CaNa_2_EDTA with BAL is recommended in Pb poisoning induced encephalopathy, while CaNa_2_EDTA can be used as a monotherapy in those patients requiring chelation but without signs of Pb induced encephalopathy [6, 45]. Compared with DMSA, CaNa_2_EDTA is better at mobilising Pb from the bone and forms a water-soluble compound that is ideal for urinary elimination and removal by EBP. Of note, combined chelation with DMSA and CaNa_2_EDTA has been shown to be more effective than either CaNa_2_EDTA or DMSA is given individually [46, 47]. However, DMSA is not appropriate for anuric patients as discussed previously.

CaNa_2_EDTA has a molecular weight of 374 Da and the molecular weight of Pb-Na_2_EDTA complex is 542 Da. CaNa_2_EDTA is hydrophilic and has a small volume of distribution of 0.21 L/kg [17]. The protein binding of EDTA in plasma is not well described, but ranges from 11 to 19% [20]. These properties make CaNa_2_EDTA and its complexes theoretically ideal for extracorporeal removal. Despite this, however, a study demonstrated that a 4-h HD session with a high-flux dialysis membrane (even if the dialysis treatment continued to infinity) could only eliminate 60 to 65% of administered 1 g CaNa_2_EDTA and the rest of the administered compound could not be accounted for [44].

In reported cases of Pb poisoning in the setting of AKI and ESKF, clinical outcomes with CaNa_2_EDTA and various EBPs are largely favourable; only one patient death has been reported, and clinical manifestations induced by Pb poisoning in the remaining 9 patients had either resolved or improved [38, 40–43]. Negative outcomes have only been reported in a single older case series. In this report, 4 paediatric patients presented with encephalopathy and extremely elevated serum Pb levels, ranging from 7.72 to 14.97 umol/L (1.6 to 3.1 mg/g) as compared with 0.63 to 3.3 umol/L in the other reported cases. Although these patients had normal renal function, the severity of their presentation led to their treatment with 50 to 75 mg/kg of EDTA and adjunctive HD. In each of these patients, only 1 to 3.4 mg of chelated Pb were removed per HD session [37], probably due to the ineffectiveness of HD which was performed using a Kolff-Brigham rotating drum artificial kidney wound with 26 loops of cellophane. Unsurprisingly, two patients died and the other 2 remained significantly encephalopathic despite treatment.

PD is generally not an ideal EBP for acute poisonings, due to its slow clearance of toxins. In chronic Pb poisoning, however, the literature shows that peritoneal dialysis can be safely used in combination with CaNa_2_EDTA so long as there is no clinical urgency [38, 40–42]. This is particularly well described in one case series: the amount of chelated Pb extracted by PD (1.48 to 2 mg) exceeded that by normal functioning kidneys (0.4 to 0.7 mg) over 20 h in 3 patients with chronic Pb poisoning; the corresponding amounts were 16.8 mg by PD and 0.12 mg by chronically diseased kidneys (eGFR < 10 ml/min) in another [38]. Critically, this approach is not recommended for acute Pb poisoning, and is only appropriate for chronic cases.

We suggest CaNa_2_EDTA in combination with BAL as the first line treatment for anuric patients with severe Pb poisoning or Pb-induced encephalopathy. If encephalopathy is not present, but chelation is needed for Pb poisoning, CaNa_2_EDTA may be used alone [6, 45]. There is categorically no role for CaNa_2_EDTA in Ars poisoning. It is difficult to determine the ideal timing of initiating EBP after administrating the chelator, although the most reports begin immediately after a one-hour intravenous infusion of CaNa_2_EDTA is completed. Longer EBP treatments will likely allow for greater removal of chelator-metal complexes.

Given the available evidence, our patient was managed with using BAL and CVVHDF as the initial treatment. This was done so to avoid worsening of encephalopathy and development of BAL related adverse effects, and for maximal removal BAL-Pb complex. He was switched to CaNa_2_EDTA and high-flux HD as soon as CaNa_2_EDTA became available, and his encephalopathy resolved. The patient responded to the combination favourably without experiencing any major adverse events and his Pb levels receded promptly. It is important to note that CaNa_2_EDTA is generally administered as a 25 – 50 mg/kg intravenous infusion over twenty four hours; however we administered 1 g over one hour prior to HD based on the literature describing those with ESKF [41, 42, 44].

### 2,3-Dimercapto-1-propanesulfonate (DMPS)

DMPS is a water-soluble analogue of BAL that has been used for heavy metals poisoning (mainly mercury, Ars and sometimes Pb) for many years. It appears to be less effective than DMSA or CaNa_2_EDTA in treating Pb poisoning [6], although good clinical outcomes have occasionally been reported [48]. There are numerous case reports showing successful treatment of Ars poisoning, as summarized in Table 3 [4, 9, 49–51].

DMPS has favourable properties for extracorporeal removal, including modest protein binding of about 62.5%, hydrophilia and a low volume of distribution (0.16 L/kg) [4, 19]. The molecular weight of its metal complex is low based on 1:1 ratio, but it could be 834 Da for DMPS-Ars compound if the compound ratio is 3:2 as has been suggested [4]. If this were the case, using high-flux HD or hemodiafiltration (HDF) would theoretically be preferable.

Adam described in his thesis a series of patients with acute Ars poisoning, including a 22 year-old patient who developed anuric AKI within 18 h of ingesting inorganic Ars. His initial serum Ars level was 4469 μg/l. He was treated with intravenous DMPS together with HDF. The maximum achieved extracorporeal clearance of Ars was 140 ml/min, and the average clearance 98 ml/min. A total of 252 mg of Ars was extracted by daily 5-h sessions of HDF over 5 days, and his serum arsenic was reduced to 106 μg/l. The patient recovered without long-term complications. Of note, this impressive clearance is still much lower than that achieved by the normally functioning kidneys, as illustrated by the measured clearance in another arsenic poisoned patient in the same report – after DMPS, clearance averaged 696 ml/min. The lowest clearance in this case series was achieved in two cases treated with BAL and combined low-flux HD plus hemoperfusion (two cases 70.4 ml/min and 125.8 ml/min) [30].

Despite the lack of definitive data, DMPS combined high-flux HD or HDF should be considered in acute Ars poisoning in auric patients. This recommendation is based on the observations of Adam above and also other reported cases [8, 10, 24]. If DMPS is given orally, EBP should be commenced within 2 h of the administrating the chelator, as the urinary excretion of Ars by normal functioning kidneys appears to be the greatest in the first 2 h [52]. Though evidence is not strong, we feel that EBP can be initiated immediately if DMPS is given intravenously, and the duration of each EBP treatment session after administrating the chelator should ideally be more than the standard 4 h to allow for greater removal of the chelator-metal complexes. In those with normally functioning kidneys, EBP probably provides little benefit.

Theoretically, DMPS could have been used as the sole and initial chelator for our patient with combined Ars and Pb poisoning. However, the severity of his Pb poisoning was such that DMPS was probably insufficient as first line chelation, even if it had been available to us at the time. It could potentially have been given in conjunction with BAL for maximizing both Ars and Pb chelation without exacerbating encephalopathy. Similarly, combining DMPS with CaNa_2_EDTA would be a preferred option in the absence of encephalopathy for their favourable chemical and pharmacological properties.

As stated previously, chelators are generally not recommended in chronic arsenicosis. Having said that, DMPS has been shown to increase urinary Ars excretion even in patients with chronic arsenicosis [52]. A small randomised placebo-controlled trial and a case report demonstrated improvement in clinical symptoms caused by chronic arsenicosis using DMPS [51, 53], although the trial was not well designed. In these reports, however, all patients had normal kidney function. In contrast, a trial of oral DMPS in our patient resulted in negligible extracorporeal removal of Ars and no significant change to his peripheral neuropathy during the course of the therapy.

## Conclusions

**Fig. 2:**
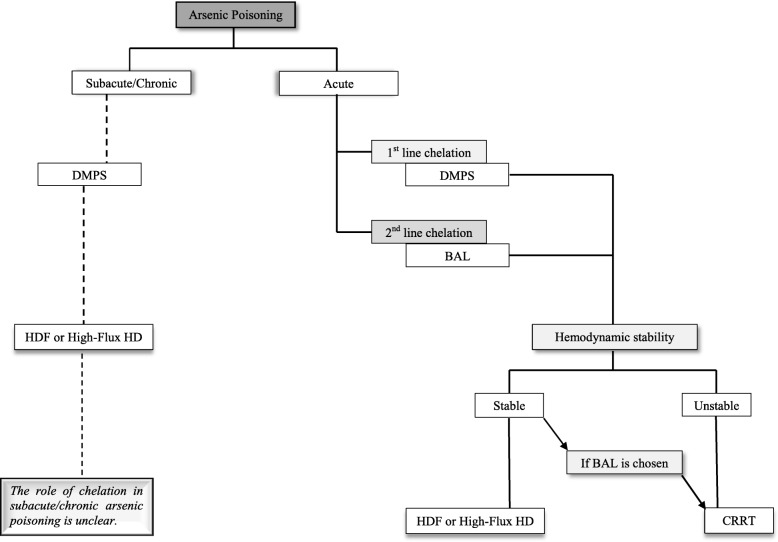
A flow chart in conjunction with Table 4 to assist treating clinicians in choosing chelation therapy and extracorporeal blood purification modality in oliguric or anuric patients with arsenic poisoning

**Fig. 3:**
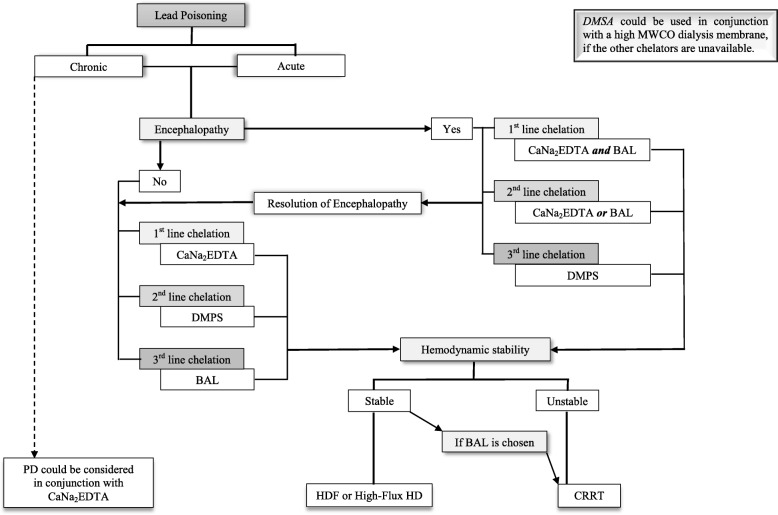
A flow chart in conjunction with Table 4 to assist treating clinicians in choosing chelation therapy and extracorporeal blood purification modality in oliguric or anuric patients with lead poisoning

Treating heavy metal poisoning in anuric patients remains a significant challenge. Based on cumulative clinical experience to date, CaNa_2_EDTA and DMPS are probably the safest and the most effective chelating agents for Pb and Ars, respectively, and DMSA should not be used in anuric patients if the other chelators are available. BAL should be used in conjunction with CaNa_2_EDTA for severe Pb poisoning or Pb-induced encephalopathy, and may be used as the sole chelator for both Pb and Ars poisoning if no other chelators are available. These suggestions are summarised in Table 4 supported by Figs. 2 and 3. High-flux HD and HDF are preferred EBP modalities, but continuous renal replacement therapy should be considered in patients with haemodynamic instability or when using BAL any time to reduce the risk of developing rebound phenomena and BAL associated adverse effects. More research in this area is needed to assist in developing evidence-based treatment guidelines for acute Pb and Ars poisoning in this population. Finally, in all cases of poisonings, treatment decisions should generally be made in conjunction with a toxicologist and/or a local poison center, if available.

**Table 4:** General guidance on chelation therapy and extracorporeal blood purification (EBP) in oliguric or anuric patients with lead and/or arsenic poisoning ^a^

Heavy metal poisoning	Chelation		EBP Modality
Acute severe Pb poisoning with encephalopathy	1st line:	BAL 4 mg/kg IM every 4-6 h [3, 22] AND CaNa_2_EDTA 25-50 mg/kg (max 3 g) IV over 24 h on CRRT, begin 4 h after BAL [41, 42, 44]	HDF or high-flux HD CRRT if unstable or BAL is used
	2^nd^line:	CaNa_2_EDTA 1 g IV over 1 h, give 1–3 h before HD or 25-50 mg/kg (max 3 g) IV over 24 h on CRRT [41, 42, 44] OR BAL 4 mg/kg IM every 4-6 h on CRRT [3, 22]	
	3rd line:	DMPS 3-5 mg/kg (max 250 mg) IV every 4 h [49–51] ^b^	
	*DMSA* ^c^		
Acute Pb poisoning without encephalopathy but still requiring chelation	1st line:	CaNa_2_EDTA 1 g IV over 1 h, give 1–3 h before HD or 25-50 mg/kg (max 3 g) IV over 24 h on CRRT [41, 42, 44]	HDF or high-flux HD CRRT if unstable or BAL is used
	2nd line:	DMPS 100-300 mg PO every 8 h [49, 52, 53]^b^	
	3rd line:	BAL 4 mg/kg IM every 4-6 hours [3, 22]	
	*DMSA* ^c^		
Chronic Pb poisoning without encephalopathy requiring chelation	1st line:	CaNa_2_EDTA 1 g IV over 1 h, give 1–3 h before HD or 25-50 mg/kg (max 3 g) IV over 24 h on CRRT [41, 42, 44]	HDF or high-flux HD CRRT if unstable or BAL is used PD could be considered if CaNa_2_EDTA is used
	2nd line:	DMPS 100-300 mg PO every 8 h [49, 52, 53]^b^	
	3rd line:	BAL 4 mg/kg IM every 4-6 h [3, 22]	
	*DMSA* ^c^		
Ars poisoning (acutely ill)	1st line:	DMPS 3-5 mg/kg (max 250 mg) IV every 4 h [49–51]	HDF or high-flux HD CRRT if unstable or BAL is used
	2nd line:	BAL 4 mg/kg IM every 4-6 h [3, 22]	
	*DMSA* ^c^		
Ars poisoning (chronic / subacute) ^d^	DMPS 100-300 mg PO every 8 h [52, 53]		HDF or high-flux HD

*CRRT* continuous renal replacement therapy, *HDF* hemodiafiltration, *HD* hemodialysis, *PD* peritoneal dialysis, *MWCO* molecular weight cut-off

^a^ The above chelation dosing recommendations are a result of our literature review and personal experience and should be considered as a guideline only. We strongly recommend discussion with a poison center or medical toxicologist in conjunction with these recommendations for each individual case, therapeutic endpoints of chelation and side-effects. 2nd and 3rd line treatments may be considered if 1st line treatments are unavailable

^b^ DMPS dose for Pb chelation is extrapolated from the Ars chelation dose

^c^ The efficacy DMSA with severe renal impairment is unclear, but it may be considered in conjunction with a high MWCO dialysis membrane if the other chelators are unavailable. For both Pb and Ars: 10 mg/kg PO every 8 h for 5 days, then 10 mg/kg every 12 h [33]

^d^ The role of chelation, if any, in chronic/subacute Ars poisoning is unclear

## Data Availability

The original de-identified data and material is available upon request from the corresponding author at chunyuan.hsiao@middlemore.co.nz.

## Abbreviations

AKI: Acute kidney injury
BAL: Dimercaprol, also known as british anti-lewisite
CaNa2EDTA: Calcium-disodium-ethylenediaminetetraacetic acid
CVVH: Continuous veno-venous haemofiltration
CVVHDF: Continuous veno-venous haemodiafiltration
DMPS: Dimercaptopropanesulfonic acid
DMSA: Dimercaptosuccinic acid
EBP: Extracorporeal blood purification
ESKF: End-stage kidney failure
HD: Hemodialysis
HDF: Hemodiafiltration
MWCO: Molecular weight cut-off
